# Bioactive VS_4_-based sonosensitizer for robust chemodynamic, sonodynamic and osteogenic therapy of infected bone defects

**DOI:** 10.1186/s12951-023-02283-6

**Published:** 2024-01-16

**Authors:** Yaqi He, Xin Liu, Jie Lei, Liang Ma, Xiaoguang Zhang, Hongchuan Wang, Chunchi Lei, Xiaobo Feng, Cao Yang, Yong Gao

**Affiliations:** 1 Department of Orthopaedics, Union Hospital, Tongji Medical College, Huazhong University of Science and Technology, Wuhan, 430022 China; 2 Department of Ophthalmology, Union Hospital, Tongji Medical College, Huazhong University of Science and Technology, Wuhan, 430022 China

**Keywords:** Ultrasound therapy, Schottky junction, Chemodynamic therapy, Vanadium tetrasulfide, High antibacterial efficiency, Osteogenesis

## Abstract

**Background:**

Most bone defects caused by bone disease or trauma are accompanied by infection, and there is a high risk of infection spread and defect expansion. Traditional clinical treatment plans often fail due to issues like antibiotic resistance and non-union of bones. Therefore, the treatment of infected bone defects requires a strategy that simultaneously achieves high antibacterial efficiency and promotes bone regeneration.

**Results:**

In this study, an ultrasound responsive vanadium tetrasulfide-loaded MXene (VSM) Schottky junction is constructed for rapid methicillin-resistant staphylococcus aureus (MRSA) clearance and bone regeneration. Due to the peroxidase (POD)-like activity of VS_4_ and the abundant Schottky junctions, VSM has high electron–hole separation efficiency and a decreased band gap, exhibiting a strong chemodynamic and sonodynamic antibacterial efficiency of 94.03%. Under the stimulation of medical dose ultrasound, the steady release of vanadium element promotes the osteogenic differentiation of human bone marrow mesenchymal stem cells (hBMSCs). The in vivo application of VSM in infected tibial plateau bone defects of rats also has a great therapeutic effect, eliminating MRSA infection, then inhibiting inflammation and improving bone regeneration.

**Conclusion:**

The present work successfully develops an ultrasound responsive VS_4_-based versatile sonosensitizer for robust effective antibacterial and osteogenic therapy of infected bone defects.

**Supplementary Information:**

The online version contains supplementary material available at 10.1186/s12951-023-02283-6.

## Introduction

Bone defects can be categorized based on their etiology into traumatic, pathological, and infectious bone defects [1]. Different from the other two types, infective bone defects have more risks such as infection spread, severe local or systemic inflammatory response, defect expansion, and difficulty in healing of the defect [1, 2]. Traditional methods for the treatment of infected bone defects include local debridement, systemic administration of antibiotics, and bone grafting [2–4]. However, improper debridement will aggravate the infection of bone defect and surrounding soft tissue, and then the expansion of wound will reduce the efficiency of healing [5, 6]. Oral or intravenous antibiotics will not only inevitably increase bacterial resistance but also cause intestinal flora imbalance and liver and kidney function damage [7–10]. Similarly, autologous bone grafting requires the destruction of normal bone tissue for harvesting which has the short supply and the considerable donor site morbidity associated with the harvest [11, 12]. In the process of bone grafting, it also faces the risk of infection, pain, and nonunion, which is difficult for many patients to tolerate [12]. Above all, a single treatment method is not sufficient to solve problems like antibiotic resistance and non-union of bones [13]. Therefore, a comprehensive and multifunctional treatment method that simultaneously achieves both high antibacterial efficiency and promotes bone regeneration is the direction of our efforts.

Nanoparticles have been widely used in resource and energy, nano electronic devices, medicine and health and biotechnology in recent years due to their good biocompatibility, low antibiotic dependence and high catalytic performance [14–17]. Conditionally responsive nanoparticles can promote drug delivery and enhance catalysis under photodynamic, photothermal, near-infrared light (NIR), microwave (MV) and ultrasound effect for the treatment of diseases such as cancer and infection [16, 18–21]. For example, near-infrared light-sensitive materials have been used to treat infected bone defects [16, 22, 23]. But for deep tissues such as bone, ultrasound-responsive nanoparticles have the advantages of non-invasiveness and high tissue penetration ability for better efficacy [19, 24]. At present, some nano-sonosensitizers have been developed, such as single atoms, chemical compounds, metal–organic framework (MOF) and titanium hydride (TiH_1.924_) nano-dots [25–28]. However, due to their single function and poor reactive oxygen species (ROS) production performance, these nanoparticles are difficult to face some complex disease environments.

VS_4_ is a semiconductor sonosensitizer, and in previous studies it was found to be a novel narrow band gap sonosensitizer [29]. Due to its narrow band gap of only 1.12 eV, electrons and holes can be easily separated under the stimulation of ultrasound (US), which has a strong potential for ROS production [30]. There have been many studies to improve the sonodynamic effect of VS_4_ by assembling VS_4_ with other substances to form heterojunctions to improve the charge utilization efficiency [31, 32]. At the same time, the peroxidase (POD)-like activity of VS_4_ also provides us with new ideas for the application scope of VS_4_ [33]. VS_4_ can not only serve as an excellent sonosensitizer, but also plays a chemodynamic role through its POD-like catalytic properties to generate many reactive oxygen species (ROS). From a biological point of view, vanadium is one of the basic trace elements in human body, which is abundant in bone and has a certain regulatory effect on energy metabolism and the growth of bones and teeth [32]. Most importantly, vanadium release from VS_4_ has great potential to promote bone regeneration [34].

As a new type of two-dimensional (2D) nanosheets obtained by etching the A layer with MAX phases, MXene has attracted more and more attention (M represents early transition metal, A represents A-group element, and X represents C and/or N element) [35, 36]. MXene mainly includes metal carbides, nitrides and carbonitrides, which gave us more space for material selection [37]. Due to its unique two-dimensional structure and good biocompatibility, MXene has been widely studied in the biomedical field for antibacterial, anti-tumor, imaging applications and so on [38–40]. The conductor-like ultra-high conductivity of MXene makes it easy for electrons to flow from the semiconductor to its surface, thus achieving a high electron–hole separation rate. In addition, the high specific surface area of MXene provides abundant attachment sites and reaction as points for semiconductors [41, 42]. As the most typical MXene, Ti_3_C_2_ has been studied to combine with many semiconductors to help it play a catalytic role in sonodynamic therapy (SDT) and chemodynamic therapy (CDT) [38, 42–44]. Although MXene is a good optional catalytic supporter, it may have limitations in mimicking the activity of peroxidase, which may affect its efficacy in therapeutic and assay applications [45].

**Scheme 1:**
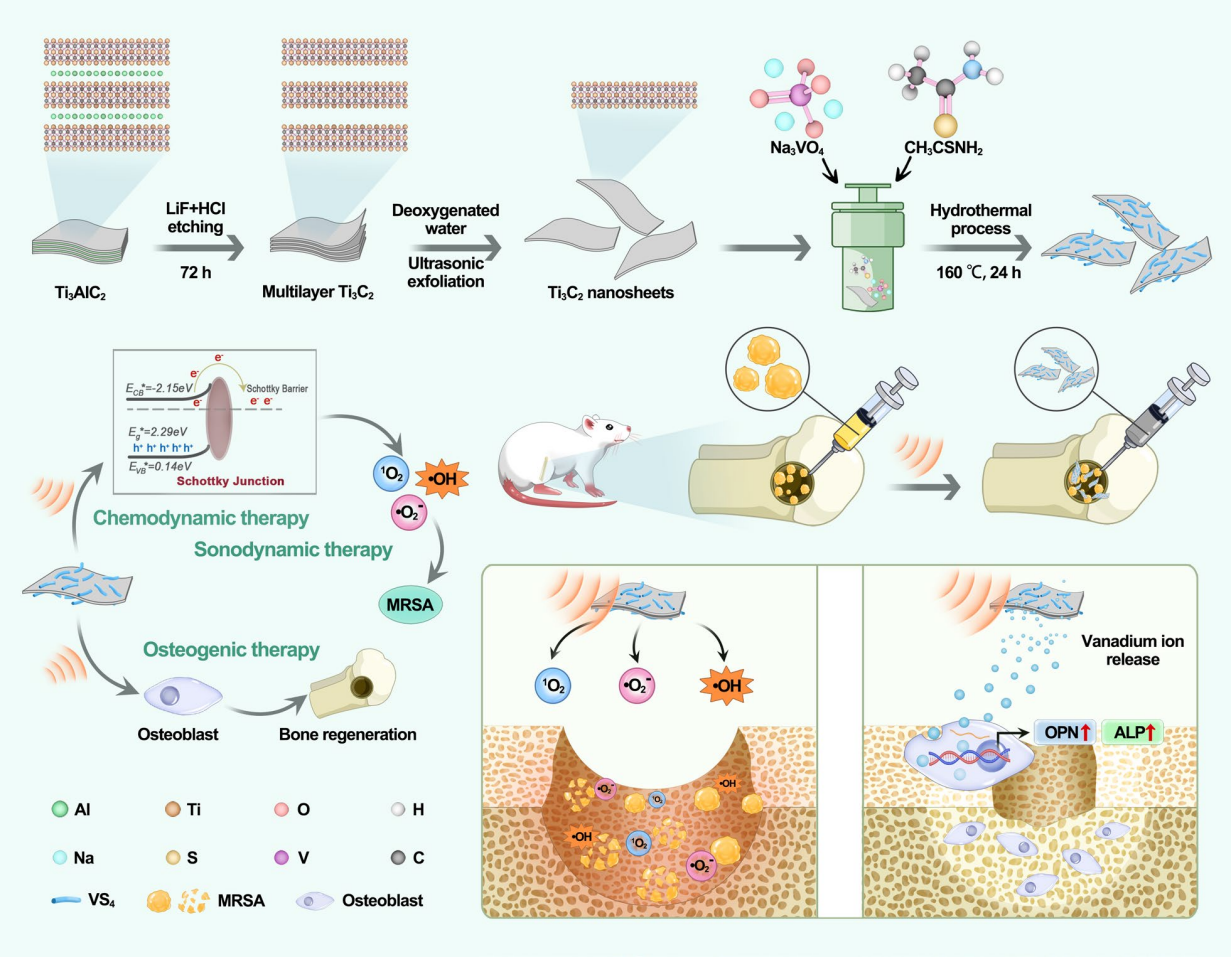
Schematic illustration of chemodynamic, sonodynamic and osteogenic therapies for infectious bone defect. Under the stimulation of ultrasound, sonosensitizer 3VSM synthesized by hydrothermal methods have high antibacterial efficiency due to the generation of large amounts of reactive oxygen species and great osteogenic ability due to vanadium release

Herein, we have synthesized VS_4_/MXene nanoparticles named VSM with Schottky junctions for efficient treatment of infectious bone defects by SDT, CDT and osteogenic therapy. VS_4_ is responsible for the major chemical properties due to its POD-like ability and sonodynamic performance, while MXene enhances the biological functions and reduced the toxicity of VS_4_. As demonstrated in Scheme 1, after simple hydrothermal synthesis with optimal ratios, VSM could exert strong antibacterial and osteogenic abilities under the stimulation of ultrasound [31]. When methicillin-resistant *Staphylococcus aureus* (MRSA) invaded the bone, VSM was stimulated by capturing the energy of ultrasound to produce abundant ROS to destroy the bacteria and clear the infection and to suppress the inflammatory response in the foci of infection and the whole body. In addition, the process of ROS generation is facilitated by the presence of MXene, which enables the rapid flow of electron–hole pairs inside VSM. MXene, acting as a two-dimensional electron acceptor, enhances the peroxidase (POD)-like ability of VS_4_. Concurrently, VS_4_ addresses the shortcomings of MXene in biocatalysis. In previous reports, vanadium ions had an exact osteogenic effect. We verified in this experiment that vanadium ions enhanced the expression of osteogenesis-related genes (*OPN*, *ALP*), which was highly consistent with our goal of treating bone defects. It is worth mentioning that VSM has achieved the more release of vanadium ions by the action of acoustoelectric current. In summary, we have designed a nanoparticle with antibacterial and osteogenic effects, which can achieve good therapeutic effect on infected bone defects with the assistance of ultrasound.

## Materials and methods

### Synthesis of VS_4_ nanorods

VS_4_ nanorods was successfully synthesized by a hydrothermal method [31, 46]. The mixed solution was obtained by dissolving 5 mmol sodium orthovanadate (Na_3_VO_4_, Macklin) and 25 mmol thioacetamide (CH_3_CSNH_2_, Macklin) in 60 mL deionized water, which pH was adjusted to 12 by 1 mol L^−1^ NaOH aqueous solution. After 0.5 h of magnetic stirring at room temperature, the solution was transferred into two 50 mL Teflon-lined stainless autoclaves and then heated to 160 °C for 24 h. After cooling to about 25 °C, the product was collected and washed at least three times with deionized water. Finally, the purified product was dried in a vacuum oven at 60 °C for 12 h to obtain VS_4_ nanorods.

### Synthesis of MXene nanosheets

Etching method was used to obtain MXene nanosheets. 2 g LiF and 40 mL hydrochloric acid (HCl, 9 mol L^−1^) solution were poured into a teflon beaker, and the mixed solution was stirred for 30 min. 2 g Ti_3_AlC_2_ was weighed and slowly added to the above solution, followed by magnetic stirring at room temperature for 24 h. The fully reacted solution was centrifuged to obtain the precipitate, which was multilayer Ti_3_C_2_. This precipitate was washed repeatedly by sonication with reverses osmosis water and finally freeze-dried to obtain monolayer Ti_3_C_2_ nanosheets, named MXene [43].

### Synthesis of VSM nanocomposites with Schottky junctions

The VS_4_/MXene nanocomposites were successfully performed by a one-step hydrothermal method [31]. Since 5 mmol sodium orthovanadate (Na_3_VO_4_, Macklin) and 25 mmol of thioacetamide (CH_3_CSNH_2_, Macklin) can generate about 0.5 g VS_4_, we set the mass ratio of VS_4_ and MXene to 1:1, 3:1, and 4:1, denominated as 1VSM, 3VSM, and 4VSM, respectively. Mixed powder in different proportions were placed in 60 mL deionized water under constant magnetic stirring until a homogeneous solution was formed at 60 °C. Then, the mixed solution was transferred into 100 mL of Teflon-lined stainless autoclave and heated to 160 °C for 24 h. After cooling to about 25 °C, the product was collected and washed with reverses osmosis water three times and placed in the refrigerator at − 20 °C for 12 h. Finally, the moisture in the heterostructure is removed by freeze–drying method, and three groups of samples 1VSM, 3VSM and 4VSM were obtained.

### Characterization of nanocomposites

The binding state and morphology of nanoparticles were observed by scanning electron microscope (SEM, Tescan Mira4, Czech Republic) and transmission electron microscopy (TEM, Thermo Fisher Talos F200s, USA). Energy dispersive X-ray spectroscopy (EDS), which was used for was elemental analysis, was performed with a spectroscope connected to TEM. The zeta potential of the nanoparticles before and after the composite was compared (Malvern Nano ZS ZEN3600, England). The crystal structure was observed by X-ray diffraction (XRD, 2 Theta from 10° to 80°, Cu Kα radiation, Rigaku SmartLab SE, Japan). X-ray photoelectron spectroscopy (XPS) spectrum was obtained (Thermo Scientific K-Alpha, United States of America) and used to analyze the chemical state of various elements in nanocomposites. The diffuse reflectance spectra were collected using ultraviolet/visible/near infrared spectrophotometer (UV–vis–NIR, Shimadzu, Japan) to detect the optical absorption characteristics of materials. Raman spectra was detected by Raman microscope (Horiba LabRAM HR Evolution, Japan). Photoluminescence (PL) spectra of the materials were acquired using a steady-state/Lifetime Spectrofluorometer (Edinburgh FLS1000, England). Each group pf materials (MXene, VS_4_, 3VSM) were immersed in 10 mL of culture medium solution with 15 mL sterile microcentrifuge tubes. After incubated for 1 ,7, 14, and 28 days, the measurement of vanadium element releasing from them were carried out by inductively coupled plasma optical emission spectrometer (ICP-OES). In addition, the US treatment was performed the day before the measurement.

### Ultrasound method

An Intelect Mobile Ultrasound equipment (Chattanooga 2776, DJO Group, United States of America) was used for all US treatment. In the test of sonodynamic effect and antibacterial property, the US parameter was 1.0 MHz, 1.2 W cm^−2^ (or 1.0 W cm^−2^), 50% duty cycle, while in experiment related to hBMSCs cells, US parameters was 1.0 MHz, 0.2 W cm^−2^, 50% duty cycle. The parameters of the US selected based on medical imaging and Rehabilitation physiotherapy [47]. A 5 mL centrifuge tube was inserted into a 5 cm couplant when ultrasound was applied to the nanoparticle dispersion or bacterial solution. For sonication of the cells, the cell culture plates were placed above couplant with a thickness of 5 cm. The ultrasonic device was all in contact with the treated object through the medicalultrasonic couplant.

### Electrochemical workstation

The acoustoelectric current of each group of samples under 1.2 W cm^−2^ US irradiation and the electrochemical impedance spectroscopy (EIS) spectra under non ultrasonic condition were measured through the electrochemical workstation (CHI660E, China).

### ROS measurement

The ability to generate ROS, including to produce hydroxyl radical (·OH), superoxide anion (·O_2_^−^) and singlet oxygen (^1^O_2_), was obtained by using electron spin resonance (ESR) and spectrophotometry. All materials were subject to US treatment before ESR testing. ESR experiments were performed on Bruker EMXplus with 5,5-dimethyl-1-pyrroline N-oxide (DMPO) and 4-amino-2,2,6,6-tetramethylpiperidinol (TEMP) as spin trapping agents for radical analysis. ·OH, ·O_2_^−^ and ^1^O_2_ were also detected by terephthalic acid (TA, NaOH solution), nitro blue tetrazolium (NBT, DMSO solution) and 1,3-diphenylisobenzofuran (DPBF, ethanol solution) respectively, using a multifunctional microplate reader to measure the absorbance or fluorescence intensity.

### In vitro antibacterial effect

The antibacterial effect of different materials (VS_4_, 1VSM, 3VSM, 4VSM) were evaluated by a spread plate method. In short, 180 µL of MRSA solution (10^9^ CFU mL^−1^) and 20 µL of nanomaterial dispersion (250 µg mL^−1^) were mixed in a 5 mL centrifuge tube. After US treatment, 20 µL solution of each group was added onto each standard Luria-Broth (LB) agar plate and then incubated for 24 h at 37 °C. In addition, we also set up a control (CTRL) group without antibacterial treatment and a US group with only ultrasound treatment. The bacterial colony numbers of six groups (CTRL, US, VS_4_, 1VSM, 3VSM, 4VSM) were counted to calculate antibacterial efficiency. The antibacterial efficiency was calculated as

1 $${Antibacterial}\; {efficiency}\; (\%)=\frac{{{A}}_{{CTRL}} - {{A}}_{{Experiment}}}{{{A}}_{{CTRL}}}\times{100\%}.$$

At the same time, the solutions under antibacterial treatment were fixed with 4% paraformaldehyde fix solution (Biosharp). After ethanol gradient dehydration, the influence of materials and ultrasound on the morphology of bacteria was observed by FE-SEM (HITACHI SU8010, Japan).

### Cell culture of hBMSCs

Human bone marrow blood was collected from patients undergoing hip surgery in Orthopedics Department of Wuhan Union Hospital, which conforms to the standards of The Ethics Committee of Tongji Medical College, Huazhong University of Science and Technology. The blood, diluted proportionally by phosphate buffered saline (PBS), was mixed with the lymphocyte separation solution (tbdscience, Tianjin). Pure human bone mesenchymal stem cells (hBMSCs) were obtained by centrifuging the mixture. DMEM/F12 (HYCEZMBIO, Wuhan, China) containing 10% fetal bovine serum (FBS, Gibco) and 1% penicillin–streptomycin was used as the culture medium for hBMSCs. All cells were cultured in culture flasks or pore plates at a cell incubator (37 °C, 5% CO_2_, 95% humidity).

### Counting kit 8 (CCK8) essay

hBMSCs were seeded in 24 well plates (2 × 10^4^ per well) and set as groups (CTRL, US, MXene+US, 3VSM+US). The cell viability was measured on day 1, 4, and 7 at the concentration of 25 µg mL^−1^. Specific experiment procedure was to remove the original medium and incubate it with 10% Cell CCK8 (HYCEZMBIO, Wuhan, China) solution for 1 h at 37 °C. 80 µL liquid from each hole was added to a 96 holes plate to measure the absorbance at 450 nm.

### Relative alkaline phosphatase activity

To determine alkaline phosphatase (ALP) activity, different groups of hBMSCs were cultured in osteogenic medium for 7 days. Cells were treated and assay solution was prepared according to the ALP assay kit (Beyotime) instructions. In brief, after treatment with lysate, the supernatant was discarded by centrifugation. Blank control, standard, and sample wells were set using 96-well plates and incubated for 10 min at 37 °C. After addition of termination solution, absorbance was measured at 405 nm.

### Real-time quantitative polymerase chain reaction (RT-qPCR)

Based on previous study, the RT-qPCR results of hBMSCs (CTRL, MXene, 3VSM, US, MXene+US and 3VSM+US groups) were analyzed to osteogenic performance. Trizol (Invitrogen, Carlsbad, CA, USA) was used to extract all RNA, and then cDNA was obtained using PrimeScriptTM RT Master Mix (TaKaRa, Japan). SYBR® Premix EX Taq™ (TaKaRa, Japan), primers and cDNA samples were mixed for a real-time florescent quantitative Polymerase Chain Reaction (PCR) detection (QuantStudio 3, Thermo, USA). The primer sequences of hBMSCs for *OPN*, *ALP* and *GAPDH* were listed (Table 1), and the relative gene expression was calculated by ^ΔΔ^Ct method.

**Table 1:** Genes and corresponding primer sequences used for RT-qPCR

Gene	Primer sequences (5′ to 3′)
*OPN*	F: TCACCAGTCTGATGAGTCTCACCATTC R: TAGCATCAGGGTACTGGATGTCAGGTC
*ALP*	F: TATGTCTGGAACCGCACTGAAC R: CACTAGCAAGAAGAAGCCTTTGG
*GAPDH*	F: TTCGACAGTCAGCCGCATCTT R: ATCCGTTGACTCCGACCTTCA

### Immunofluorescent staining

The stem cells were seeded in the climbing slices of 6-well plates and cultured with osteogenic medium with or without nanoparticles. The cells were divided into CTRL, US, 3VSM and 3VSM+US groups for corresponding treatment. After 14 days, cells were evenly distributed on the slides, and the slides were removed, washed 1–2 times with PBS, fixed with 4% paraformaldehyde for 30 min, and then washed again with PBS after fixation. Cells were permeabilized with 0.5% Triton x-100 at room temperature and blocked for 30 min. After the blocking solution was removed by aspiration, appropriate amounts of osteopontin (OPN, rabbit source, AF0227) and ALP (rabbit source, DF6225) antibodies were added to the wells. Antibodies were discarded after 12 h of incubation at 4 °C and washed with 0.1% phosphate buffered saline tween (PBST). Red fluorescent anti-rabbit antibody (Proteintech, SA00013-4) was added to each well and incubated for 1 h at room temperature in the dark. The anti-rabbit antibody was discarded and washed with PBST. The cytoskeleton was stained with green fluorescent solution of phalloidin solution (Yeasen, 40735ES75), and the nucleus was stained with 4′,6-diamidino-2-phenylindole solution (DAPI, Beyotime, P0131). Finally, fluorescence staining images were taken by a fluorescence microscope (Olympus IX71, Tokyo, Japan).

### Alizarin red S (ARS) staining

For ARS staining experiment, groups of hBMSCs were cultured in osteogenic differentiation medium for 14 or 21 days. After washing with PBS and fixing with 4% paraformaldehyde, cells were stained with 0.2% Alizarin red S solution (Solarbio), and stained cell images were captured by an optical microscope (Nikon H600L, Tokyo, Japan).

### H_2_O_2_ reduction test

hBMSCs were co-cultured with 100 µg mL^−1^ H_2_O_2_ for 24 h, then the medium was replaced, and the cells were treated with ultrasound and nanoparticles accordingly. Cells were harvested 1 day later and lysates were added at a ratio of 100 µL of hydrogen peroxide detection lysate (Beyotime) per 10^6^ cells, followed by sufficient homogenization to break up and lyse the cells. After centrifugation, the supernatant was used for subsequent determinations. A standard H_2_O_2_ concentration curve was prepared after calibration of the hydrogen peroxide standard. 100 µL samples and 100 µL hydrogen peroxide detection reagents were added to one assay well. Mix wells with shaking and leave for 30 min at room temperature. Then the absorbance at 560 nm was measured immediately and compared with the standard curve to calculate the concentration of H_2_O_2_ in the samples.

### Animal model and treatment

The Institutional Animal Care and Use Committee (Tongji Medical College, Huazhong University of Science and Technology, Wuhan) approved the animal experiment ethics (IACUC number: 2821). Male Sprague-Dawley (SD) rats weighing approximately 350 g, which were purchased from the Laboratory Animal Center of Huazhong University, were selected as experimental animals. Thirty rats were randomly divided into six groups: normal, control (CTRL), US, vancomycin (VAN), T (only 3VSM injection, without bacterial infection), 3VSM+US. Initially, thorough disinfection of the instruments and rat skin was conducted using iodine solution. After anesthetization with 3% phenobarbital sodium, the right tibial plateau was visible because of the cutting of skin, subcutaneous fascia, and muscle. Then, an electric drill was used to drill a hole (1.5 mm diameter) in the right tibial plateau. MRSA solution (10^9^ CFU mL^−1^, 100 µL) was injected into the hole except normal group and then the bone defect was sealed with bone wax. After establishing the infected bone defect model, various treatments were administered based on aforementioned-groups. 200 µL nanomaterial solution (25 µg mL^−1^) was injected into the defect site in 3VSM+US group and T group. In the VAN group, vancomycin solution (40 mg kg^−1^) was injected into the rats through the tail vein. The US treatment (1.0 MHz, 1.2 W cm^−2^, 50% duty cycle, 10 min) was administered to the defect site on postoperative days 1, 7, 14, 21, and 28. All rats were killed on postoperative day 28 to evaluate the osteogenic performance and the level of inflammation.

### Micro CT analysis

The right femoral specimens were scanned using a BRUKER Micro-CT SkyScan 1176 imaging system. The computed tomography (CT) images were reconstructed and analyzed by SkyScan CT-Analyser software. The 3D reconstruction of VOI was done with 3D viewer (Microsoft Corporation). Technical support for this experiment was provided by the Huazhong University of Science and Technology & Technology Analytical & Testing Center, Medical sub-center.

### Histological analysis

Tibia samples were all decalcified for 28 days before sectioning. The decalcified sections were stained with Masson, Safranine O-Fast Green, Hematoxylin–eosin (HE) and Giemsa. An optical microscope (Nikon H600L, Tokyo, Japan) was used to observe sections after staining.

### Statistical analysis

We use the mean ± standard deviation to show our data, and all experiments were performed at least three times. The resulting data were analyzed using GraphPad Prism 9 or Origin 2021 software. In addition, Student’s t-test, one-way ANOVA, or two-way analysis of variance based on analysis of variance were used to assess for significant differences between group means. *P < 0.05, **P < 0.01 and ***P < 0.001 were considered statistically significant, n.s. stands for not significant.

## Results and discussion

### The selection of final synthesis ratio

**Fig. 1:**
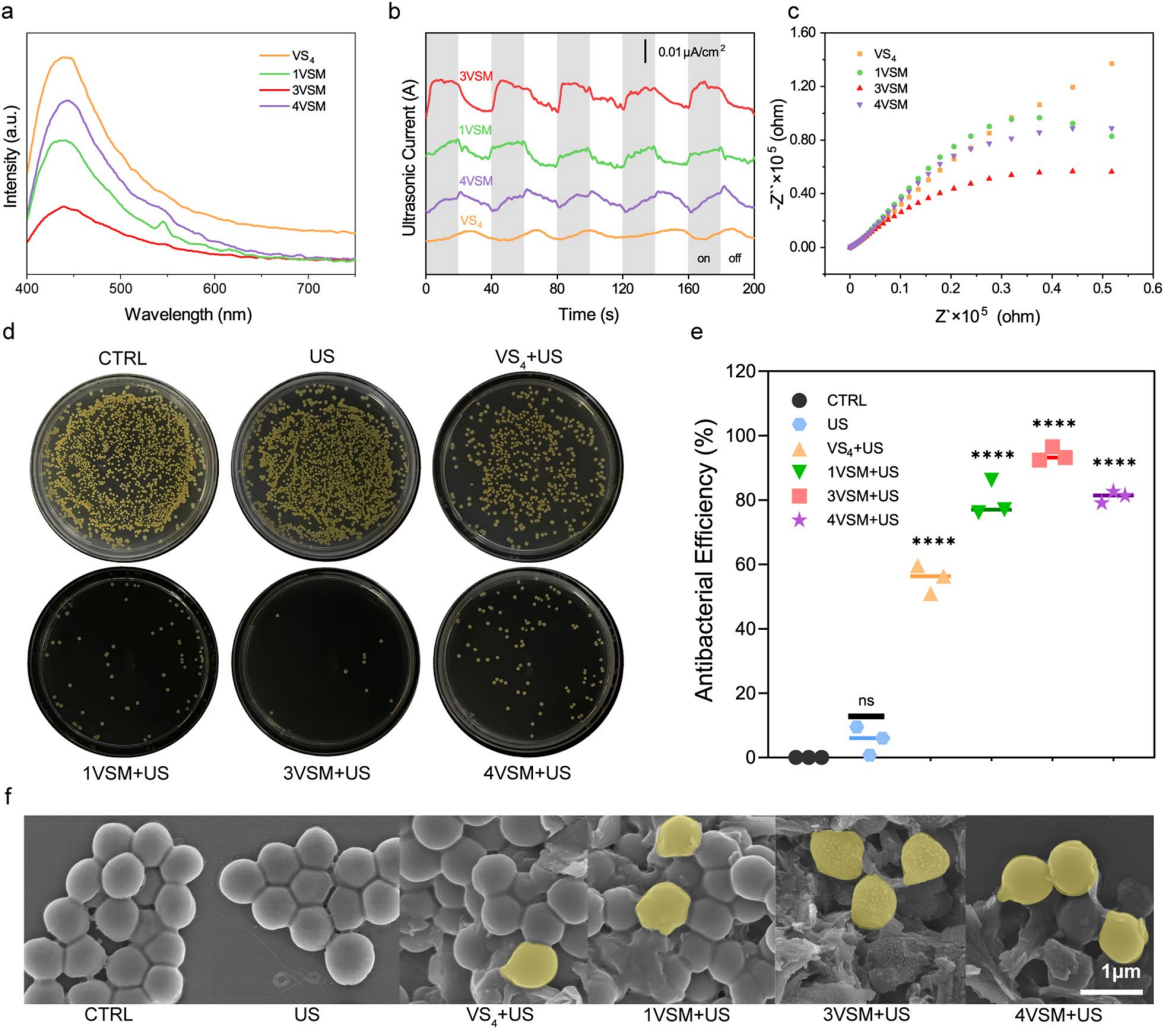
The selection of final synthesis ratio. **a** Photoluminescence spectra of VS_4_, 1VSM, 3VSM, and 4VSM. **b** acoustoelectric current test of VS_4_, 1VSM, 3VSM and 4VSM. **c** Electrochemical impedance measurement of VS_4_, 1VSM, 3VSM and 4VSM. **d** Spread plate, **e** the number of MRSA colonies and **f** FE-SEM of MRSA of CTRL, US, VS_4_+US, VSM+US, 3VSM+US and 4VSM+US. n = 3 independent experiments per group, *P < 0.05, **P < 0.01, ***P < 0.001, n.s. stands for not significant

The ultrasonic effects of VSM with different composite ratios of VS_4_ and MXene named 1VSM, 3VSM, and 4VSM were compared. Firstly, the sonocatalysis activity of VS_4_ and VSM was measured by photoluminescence (PL) spectra (Fig. 1a). We can see that 3VSM has the lowest fluorescence intensity, followed by 1VSM and 4VSM, and VS_4_ alone has the highest spectral line, which indicated that 3VSM has the highest electron–hole separation efficiency.

In order to further explore the sonodynamic catalytic properties of the samples, the acoustoelectric current response and electrochemical impedance spectroscopy were measured under US (1.2 W cm^−2^, 50% duty cycle, 1.0 MHz). As shown in Fig. 1b, the ultrasonic current of the composite nanoparticles was significantly higher than that of the VS_4_ alone, and the intensity of the acoustoelectric current is 3VSM > 1VSM > 4VSM in different composite ratios, which indicates that the 3VSM has the highest electron–hole separation efficiency under the excitation of ultrasound. From the electrochemical impedance spectroscopy results (Fig. 1c), according to the size of the semicircle arc, the electron transfer resistance is sorted as 3VSM < 1VSM < 4VSM, which is consistent with the results of the acoustoelectric current. In addition, we tested the temperature changes with infrared thermal imaging in the process of antibacterial. In the presence of ultrasound, the addition of different concentrations of nanoparticles had little effect on the temperature in bacteria solution and none of them were above 50 °C (Additional file 1: Fig. S1). According to this, temperature changes can be ruled out from impact factors of antibacterial efficiency.

In order to verify that 3VSM had the strongest antibacterial ability under US stimulation, the final concentration of the four groups of materials was set to be 25 µg mL^−1^, which was the minimum antibacterial concentration of nanoparticles. Ultrasonic responsiveness, antibacterial performance and ROS generation abilities were tested respectively. First, the in vitro antibacterial ability of nanoparticles was determined by spread plate experiments (Fig. 1d). The antibacterial efficiency (Fig. 1e) was 3VSM (94.03%) > 4VSM (81.07%) > 1VSM (79.77%) > VS_4_ (55.63%), which is consistent with the above results of PL spectra and ROS generation tests. In addition, the bacterial clearance efficiency of US group was 5.4% which proved that ultrasound alone has little antibacterial effect. The field emission scanning electron microscopy (FE-SEM) results (Fig. 1f) showed that the morphology of bacteria in control (CTRL) group and US group had little change, while the 3VSM+US treatment caused the most serious damage to the morphology of bacteria. Dead bacteria with obvious wrinkle and breakage were marked yellow. Additionally, when the powdered VS_4_ and VSM were successfully synthesized, a part of the powder was stored in sealed container and placed in a cool dry place. After 6 months, we tested its antibacterial efficiency using the same spread plate experiments. The results showed that the powder material still maintained high antibacterial efficiency (Additional file 1: Fig. S2). Among them, the antibacterial efficiency of 3VSM was 94.37%, which is highly consistent with the results of previous experiments. The maintenance of high antibacterial efficiency means that VS_4_ and VSM have high stability, which helps to exert therapeutic effect in the long term.

**Fig. 2:**
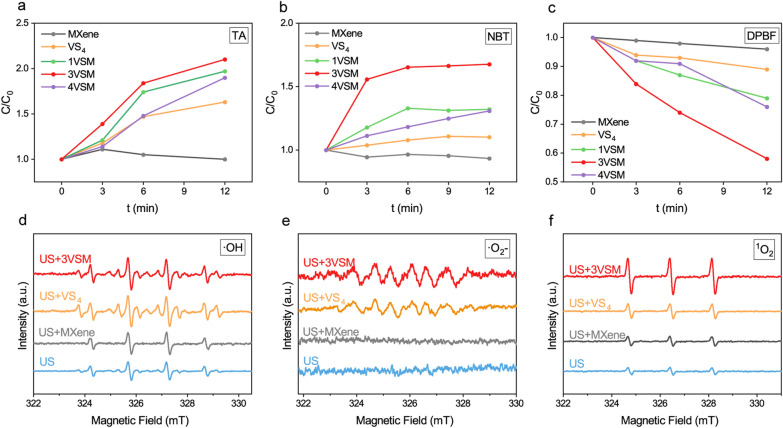
ROS generation ability and in vitro antibacterial performance. **a** ·OH generation of using TA method. **b** The generation of ·O_2_^−^ using NBT method. **c** ^1^O_2_ generation of DPBF method; **d** ESR measurements of ·OH of VS_4_, MXene and 3VSM. **e** ESR measurements of ·O_2_^−^ of VS_4_, MXene and 3VSM. **f** ESR measurements of ^1^O_2_ of VS_4_, MXene and 3VSM. n = 3 independent experiments per group, *P < 0.05, **P < 0.01, ***P < 0.001, n.s. stands for not significant

To directly prove whether nanoparticles can produce reactive oxygen species (ROS) under US radiation, the generation of ·OH, ·O_2_^−^, and ^1^O_2_ were detected using spectrophotometry analysis first by terephthalic acid (TA), nitro blue tetrazolium (NBT) and 1,3-diphenylisobenzofuran (DPBF,), respectively (Fig. 2a–c). The experiment was divided into five groups (MXene, VS_4_, 1VSM, 3VSM, 4VSM). For ·OH detection, the changes at 425 nm of TA were recorded every 3 min. With the increase of US radiation time, the fluorescence intensity of 3VSM was the strongest, which showed the highest ·OH generation. The production of ·O_2_^−^ was further verified by NBT degradation assay. Which was different from TA assay, samples were diluted with dimethyl sulfoxide (DMSO) and the fluorescence was observed at 525 nm. The results of NBT were highly consistent with TA. DPBF was a fluorescent molecule whose fluorescence intensity decreased after reaction with ^1^O_2_^**.**^ 3VSM had the most drastic decrease in fluorescence intensity, meaning that its ability to produce ^1^O_2_ was the strongest. Then, the ability of samples to produce the above three ROS was further verified through electron spin resonance (ESR) spectroscopy analysis (Fig. 2d–f). The results showed that 3VSM had the highest ROS signal peaks, which was consistent with previous experiments. ROS generation illustrated that 3VSM is superior to other groups due to the highest electron–hole separation rate.

In order to confirm the POD-like capacity of VS_4_, hydrogen peroxide (H_2_O_2_) concentration measurements were made during cell culture (Additional file 1: Fig. S3). In the presence of VS_4_ and VSM, the content of H_2_O_2_ in the hBMSCs culture environment was significantly reduced. This is due to the oxidization of hydrogen peroxide to hydroxyl radicals by VS_4_.

### Morphology and chemical composition characterization of nanoparticle

**Fig. 3:**
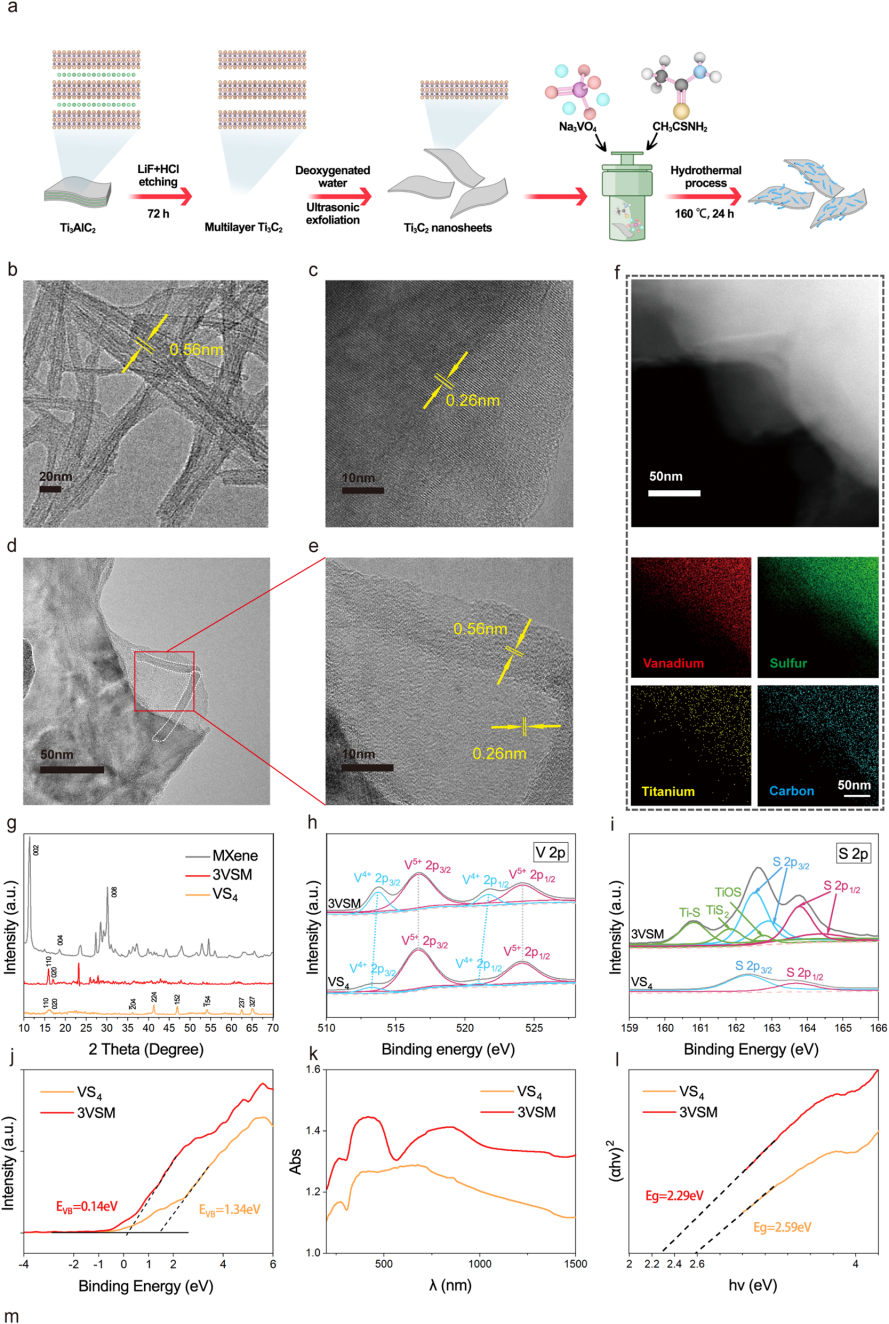
Fabrication procedure and chemical characterization. **a** Synthesis process of VSM. Ti_3_C_2_ nanosheets were obtained by etching Ti_3_AlC_2_ through LiF and hydrochloric acid. Na_3_VO_4_ and CH_3_CSNH_2_ were mixed into Teflon-lined stainless autoclaves and then heated to 160 °C for 24 h to form VS_4_ nanorods. The mixture of VS_4_ and Ti_3_C_2_ were successfully performed to obtain VSM heterostructure. TEM images and lattice spacing of **b** VS_4_, **c** Ti_3_C_2_. **d** 3VSM, **e** Schottky junction; **f** high-Angle Annular Dark Field (HAADF) scanning TEM images and corresponding area-element mapping of V, S, C, Ti in 3VSM using high resolution transmission electron microscopy. **g** XRD patterns of VS_4_, MXene and 3VSM. Energy-dispersive X-ray spectroscopy elemental mapping of V and S in VS_4_ and 3VSM. **h** V 2p spectra of VS_4_ and 3VSM. **i** S 2p spectra of VS_4_ and 3VSM. **j** valence band of VS_4_ and 3VSM. **k** UV–vis adsorption spectrum of VS_4_ and 3VSM. **l** band gap energy of VS_4_ and 3VSM

The synthesis process of MXene nanosheets, VS_4_ nanorods, and VSM nanocomposites are shown in Fig. 3a briefly. Transmission electron microscope (TEM) of VS_4_ in Fig. 3b exhibited the rod-like structure of VS_4_ with length around 50–200 nm and lattice fringe of 0.56 nm correspond to (110) plane [48]. Figure 3c showed images of the nanosheet structure of MXene from TEM. The size of MXene is 1–3 μm, and moreover, the obvious lattice stripes with a spacing of 0.26 nm is in good agreement with (010) crystal plane of Ti_3_C_2_ [49]. Scanning electron microscopy (SEM) images demonstrated the rod-like structure of VS_4_ and the lamellar structure of MXene more clearly (Additional file 1: Fig. S4a, b). From the image of 3VSM in Fig. 3d, the nanorods was loaded on the surface of nanosheets. From Fig. 3e, the heterogeneous interface of VS_4_ (0.56 nm) and Ti_3_C_2_ (0.26 nm) can be observed distinctly, demonstrated that the heterojunction of VSM was successfully constructed. As shown in Fig. 3f and Additional file 1: Fig. S4c, the energy dispersive spectrometer (EDS) element mapping clearly showed the distribution and content of all elements on 3VSM. The mean zeta potentials of VS_4_ and Ti_3_C_2_ were − 50.63 eV and − 42.8 Ev, respectively. The zeta potential of 3VSM was − 47.73 eV after VS_4_ and Ti_3_C_2_ were combined. The change of zeta potentials also indicated the success of combination of VS_4_ and Ti_3_C_2_ (Additional file 1: Fig. S5a). The results of XRD are shown in Fig. 3g. The diffraction peaks of monoclinic VS_4_ at 15.80°, 17.00°, 36.51°, 47.92°, 49.13°, 53.96°, 62.40°, 65.06° are assigned to the (110), (020), ($\bar{2}$04), (224), (152), ($\bar{1}$54), ($\bar{2}$37) and ($\bar{3}$27) planes [48]. The obvious diffraction peaks of MXene around 6.9°, 14.7°and 29.8° can be assigned to (002), (004) and (008) facets of Ti_3_C_2_ nanosheets [50]. The VSM contained the main characteristic peaks of VS_4_, which showed that the combination of VS_4_ and MXene has little effect on the crystallinity of VS_4_ nanoparticles.

X-ray photoelectron spectroscopy (XPS) was used to characterize the elements of V, S, Ti, and C of samples. Peak-differentiating and imitating of the chemical states for the individual elements was performed in MXene and 3VSM using XPS data. As shown in Additional file 1: Fig. S5b, no signal for MXene was detected in 3VSM, indicating a high loading of VS_4_. As for individual element analysis, we first analyzed the spectral peaks of vanadium element (Fig. 3h). Peak shifts of V^4+^ and V^5+^ could be seen, which manifested that the electrons around vanadium have changed because of the attachment of MXene. In S 2p mode, new peaks presented at 160.8, 161.9 and 162.8 eV, representing the existing of Ti–S and Ti–O–S bond (Fig. 3i). In Ti 2p mode, in addition to the Ti–C peaks at 454.9 and 461.0 eV, a small number of Ti–O peaks at 456.6 and 462.9 eV and Ti–F peak at 458.3 eV were detected, due to a handle of MXene that was not etched completely (Additional file 1: Fig. S5c). Figure 3j showed the valence band from XPS data analysis, which demonstrated that the valence band energies of VS_4_ and 3VM are 1.34 eV and 0.14 eV, respectively. The reduction of the valence band implied that 3VSM possesses a stronger reducing action than VS_4_. UV–vis absorption spectrum was used to investigate the light absorption characteristics of samples. Figure 3k showed that the UV–vis absorbance of 3VSM was enhanced compared with VS_4_ range from 200 to 1500 nm. Next, Kubelka–Munk diagrams of VS_4_ and 3VSM (Fig. 3l) were made based on UV–vis absorbance spectra. The band gaps of VS_4_ and 3VSM were obtained to be 2.59 eV and 2.29 eV, respectively, demonstrating that the band gap was significantly reduced after the formation of Schottky junction. Additional file 1: Figure S5d showed the Raman spectra of VS_4_, MXene and 3VSM in the range of 20–1400 cm^−1^. The peaks observed at 191 and 218 cm^−1^ are indicative of the stretching modes of V–S bonds. Additionally, the peaks found at 285, 543, and 557 cm^−1^ are associated with the stretching and twisting of S–S bonds [51]. The Raman spectra also revealed distinct characteristic peaks for Ti_3_C_2_ at 414 cm^−1^ and 600 cm^−1^, aligning with previous results. After VS_4_ loaded to MXene, there was no significant changes in the main characteristic peaks, which proved the successful preparation of 3VSM.

The work emitted by a semiconductor is generally smaller than that of a conductor, so when the conductor is in contact with the semiconductor, electrons flow from the semiconductor into the conductor. A space charge region composed of positively charged immobile impurity ions was formed in the surface layer of the semiconductor. In this region, there is an electric field directed by the semiconductor to the metal, which acts to prevent the electrons in the semiconductor from continuing to flow into the conductor. At the interface of the 3VSM, the energy band of the semiconductor VS_4_ bends to form a high potential energy region, which is the Schottky barrier (Additional file 1: Fig. S5e). Electrons in the energy band with rectifying properties continuously flowed from VS_4_ to MXene, reducing the energy required when VS_4_ catalyzed reactions. It is precisely because 3VSM has the highest saturation that 3VSM has the highest catalytic efficiency.

### In vitro osteogenesis and mechanism

**Fig. 4:**
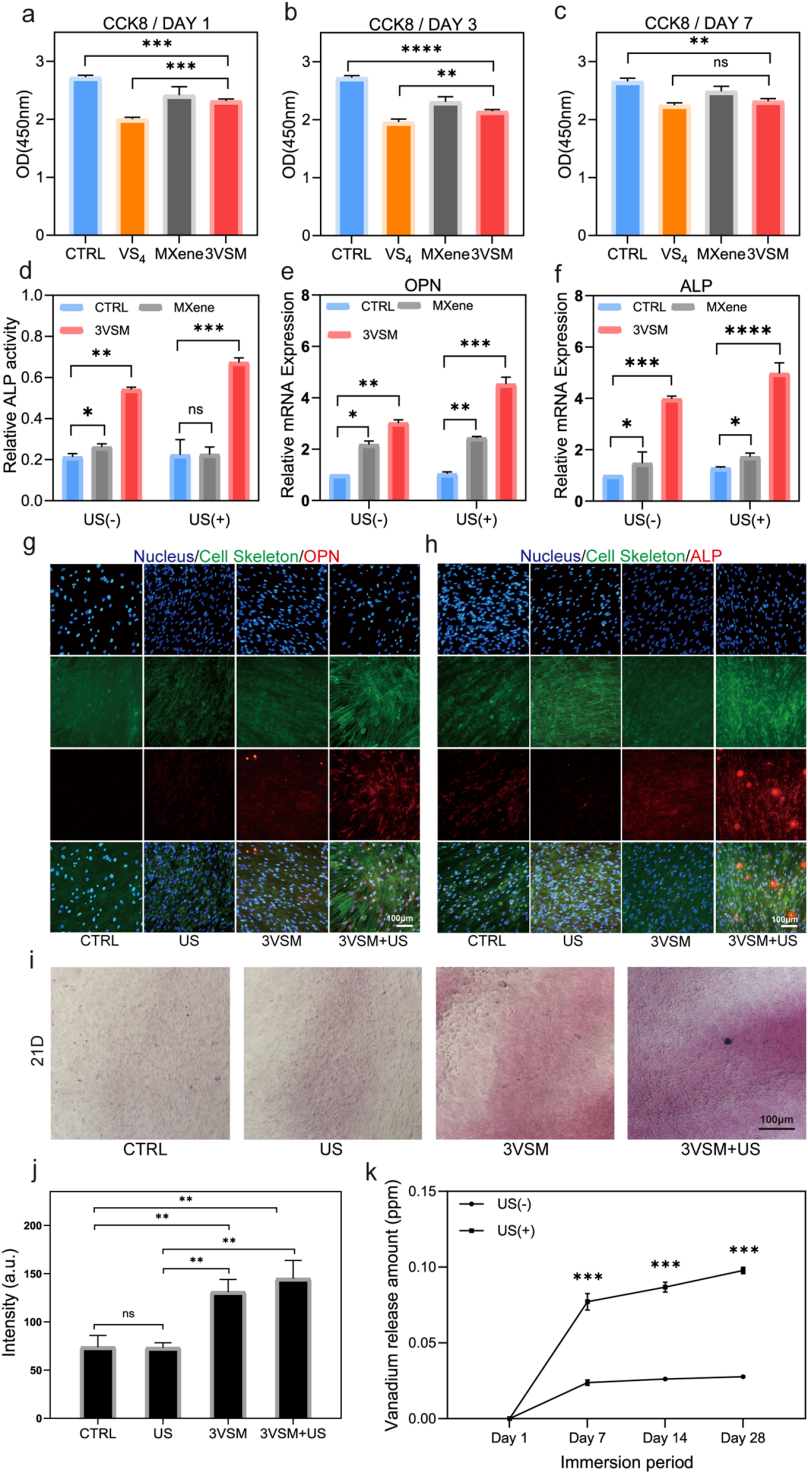
Osteogenesis of 3VSM in vitro and osteogenic mechanism. the cyto-compatibility of VS_4_ and 3VSM tested by CCK-8 method in **a** day 1, **b** day 3 and **c** day 7. **d** relative ALP activity after 21 days of CTRL, MXene, 3VSM, US, MXene+US, 3VSM+US. RT-qPCR results about **e** OPN and **f** ALP in groups of CTRL, MXene, 3VSM, US, MXene+US, 3VSM+US. **g** ALP and **h** OPN immunofluorescence staining images of hBMSCs after 21 days in different groups: CTRL, 3VSM, US, 3VSM+US. **i** ARS staining images and **j** quantitative analysis of hBMSCs after 21 days in different groups: CTRL, 3VSM, US, 3VSM+US. **k** Vanadium element release of 3VSM on day 1, 7, 14 and 28 with or without US treatment. n = 3 independent experiments per group, *P < 0.05, **P < 0.01, ***P < 0.001, n.s. stands for not significant

Before verifying the osteogenic properties in vitro, the biocompatibility of the materials was first investigated. Counting Kit 8 (CCK8) assay on day 1, 3 and 7 (Fig. 4a–c) proved that VS_4_ was slightly toxic to hBMSCs, while loading on MXene reduced the inhibitory effect on cell viability. Finally, 3VSM showed good biocompatibility, so it can be considered as a form of green materials. Figure 4d showed the analysis result of quantified ALP activity of hBMSCs, which were cultured with nanoparticles under US or without US treatment. In addition, US helped to enhance this activity. Next, the transcriptional levels of several osteogenesis-related genes were verified by real-time Quantitative Polymerase Chain Reaction (RT-qPCR) in Fig. e,f. Both *OPN* and *ALP* are key genes related to osteogenesis. *OPN* can promote the mineralization and absorption of bone matrix, and *ALP* is an early osteogenic marker that can promote the maturation of osteocytes. It was proved that 3VSM contributed a lot to the expression of osteogenic genes owing to the release of vanadium. Apparently, osteogenesis was stronger after US treatment, which was attributed to the promoting effect of US on vanadium release. The expression of osteogenesis-related proteins (ALP, OPN) was verified by immunofluorescence (Fig. 4g, h). By comparing the fluorescence patterns before and after nanoparticles and before and after the nanoparticles were stimulated by US, it was clear that 3VSM promoted the expression of osteogenic proteins. At the same exposure time, the fluorescence intensity of osteogenic proteins around the cells was higher after the addition of 3VSM. Similarly, US treatment of 3VSM enhanced the protein expression. In addition, the formation of cytoskeleton was also promoted by nanoparticles. Alizarin red S staining (Fig. 4i and Additional file 1: Fig. S6) was used to observe the mineralization of osteoblasts at 14 and 21 days, and the quantitative analysis of ARS staining at 21 days was demonstrated on Fig. 4j using Image J software. The results showed that 3VSM under US radiation had the deepest staining and the richest mineralized nodes.

To explain the mechanism of osteogenesis, previous studies on the osteogenesis of vanadium compounds were first reviewed which explained that vanadium ions could regulate osteogenic differentiation of hBMSCs through the activation of the *Itga 2b–FAK–MAPK (pERK1/2)* signaling pathway [34, 52]. The release of vanadium ions was increased by MXene loading and US treatment. As shown in Fig. 4k, vanadium ions which were released from 3VSM entered the cells, up-regulated the expression of ALP and OPN, then promoted bone regeneration. It is worth noting that ultrasound markedly enhance the release of vanadium.

### In vivo biological functions

**Fig. 5:**
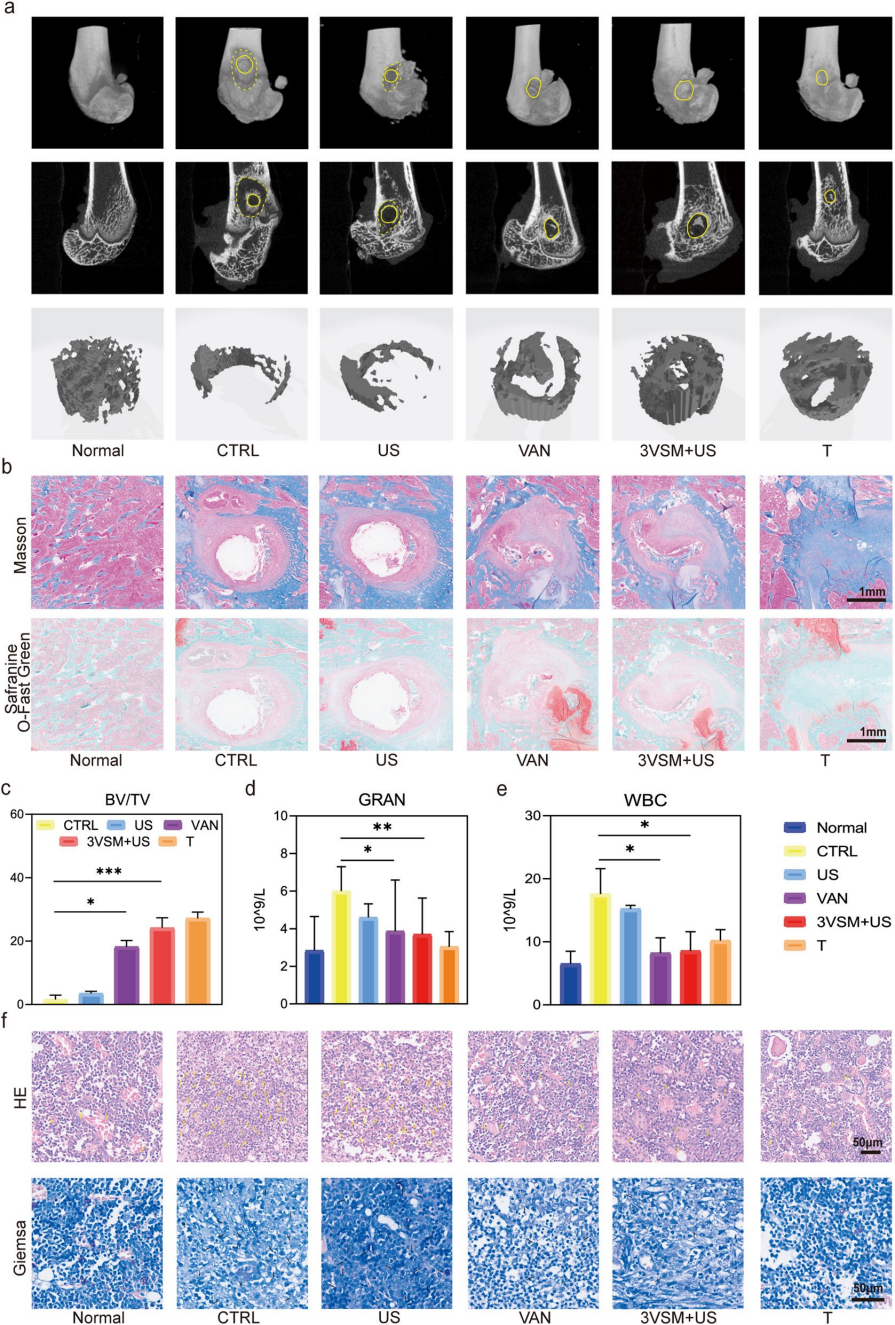
Treatment of infectious bone defect and verification of material functions. SD rats was put to death after 28 days of treatment, and **a** Reconstructed 3D images (first row), single-layer CT images (second row) and 3D picture of VOI (third row) processed from Micro-CT data. **b** Masson staining (first row) and Safranine O-Fast Green staining (second row) of samples; **c** quantitative analysis of new bone BV/TV. **d**, **e** SD rats on day 7 after surgery for WBCs and nGRANs test. **f** HE staining (first row) and Giemsa staining (second row) of bone samples. n = 3 independent experiments per group, *P < 0.05, **P < 0.01, ***P < 0.001, n.s. stands for not significant

The in vivo antibacterial effect and osteogenic ability were validated using a tibial plateau infected bone defect model. First, the specimens on day 28 after surgery were scanned by micro computed tomography (Micro-CT), and the scanning images were processed by software. First, the data were reconstructed into three dimensional (3D) images (Fig. 5a, the first row). The defect pores were marked with solid yellow lines, and the area of obvious bone destructions were marked with dashed yellow lines. Compared with CTRL, the smallest bone defect pores were in 3VSM because of no infection, followed by 3VSM+US and vancomycin (VAN) groups, while CTRL and US could see obvious bone destruction around the defect pores. Single-layer CT images (Fig. 5a, the second row) more clearly demonstrated the extent of bone destruction from the cross section, with no significant defect enlargement in 3VSM and VAN, while CTRL and US showed severe bone loss around the defect holes due to infection. The rat killed immediately after surgery was set as the standard volume of interest (VOI), and the 3D reconstruction of all the defect holes was performed to study the new bone growth using this data. According to the reconstructed 3D picture of VOI (Fig. 5a, the third row), T had the largest volume of new bone, followed by 3VSM, and the new bone showed an obvious trend of inward growth from the edge of the defect hole, with dense bone. The femur specimens were then decalcified and sectioned for staining. In Masson staining (Fig. 5b, the first row) collagen fibers were blue and muscle fibers were red, while in Safranine O-Fast Green staining (Fig. 5b, the second row), bone was green and cartilage was red, so the content of collagen fibers and bone further confirmed the osteogenesis described above. In Fig. 5c, yellow arrows in hematoxylin–eosin (HE) staining indicate inflammatory cells with lobulated nuclei and black arrows in Giemsa staining indicate positive bacteria. It could be seen, the in vivo antibacterial and anti-inflammatory effects of 3VSM+US and VAN were similar. At the same time, the reduction of inflammatory cells in the results of HE staining also proved the reduction of oxidative stress in vivo.

The biological functions were quantified as follows. The analysis of new bone in Fig. 5d confirmed the above results in Fig. 5a and b. In particular, the new bone content in 3VSM defect holes was better than that in VAN, showing excellent treatment effect of infectious bone defect. Similarly, the antibacterial and anti-inflammatory effects in vivo of VSM were validated in the above animal experiments. First, blood was drawn from caudal vein in SD rats on day 7 after surgery for white blood cells (WBCs) and neutrophil granulocytes (GRANs) examination (Fig. 5e, f). Compared with the normal rats (Normal), CTRL had the highest WBC and GRAN counts and the most severe infection. It was negligible that US treatment slightly inhibited the growth of WBC and GRAN. VAN and 3VSM+US inhibited GRAN and WBC growth significantly, slightly higher than normal. The results of blood routine showed that the systemic antibacterial effect of 3VSM+US was about equally to vancomycin intravenous injection.

## Conclusion

In summary, we have created Schottky heterojunctions between VS_4_ and MXene, which has antibacterial and osteogenic properties through chemodynamic, sonodynamic and osteogenic effects to achieve a comprehensive treatment of infectious bone defects. Under the stimulation of ultrasound, abundant electrons generated by VS_4_ were captured by MXene, and the high electron–hole separation rate achieved a high production of three types of ROS and a strong antibacterial ability, in particular the POD-like performance to generation ·OH of VS_4_. This process is further facilitated by the large influx of electrons from VS_4_ into MXene, benefiting from the formation of Schottky junctions. Vanadium ions could be released controllably under US irradiation to improve the osteogenesis ability of hBMSCs. The anti-infection and the control of inflammation played a good auxiliary role in osteogenesis. Therefore, this study simultaneously solved the typical problems of infected bone defects, and provided a non-invasive, precise, and comprehensive treatment strategy for bone infection diseases which can replace the role of antibiotics. Like other powdered nanomaterials, 3VSM might face biomechanical limitations when addressing extensive long bone defects. With this limitation, the future integration of 3VSM with implant scaffolds promises substantial advancements in this field [53].

### Supplementary Information

**Additional file 1: Figure S1.** Temperature changes during antibacterial treatment which was observed with a thermal imager. **Figure S2.** Antibacterial efficiency of materials after 6 months of placement. **a** Spread plate, **b** the number of MRSA colonies. **Figure S3.** Hydrogen Peroxide Assay Kit was used to test the H_2_O_2_ reduction rate, which was mainly benefit by the Pox-like activity of VS_4_. **Figure S4.** Materials morphology and EDS analysis.**a **TEM of VS_4_ nanorods; **b** TEM of MXene nanosheets. **c** EDS analysis of 3VSM. **Figure S5.** Characterization of nanoparticles.** a** Zeta potential of VS_4_, MXene and 3VSM. **b** The survey XPS spectra of VS_4_, MXene and 3VSM. **c** XPS spectrum in Ti 2p mode. **d** Raman spectra of VS_4_, MXene, and 3VSM. **e** Schottky junction mechanism. **Figure S6.** ARS staining of hBMSCs on day 14.

## Data Availability

The data that support the findings of this study are available from the corresponding author upon reasonable request.
