# Correction: Bioactive VS4-based sonosensitizer for robust chemodynamic, sonodynamic and osteogenic therapy of infected bone defects

**DOI:** 10.1186/s12951-024-02303-z

**Published:** 2024-01-31

**Authors:** Yaqi He, Xin Liu, Jie Lei, Liang Ma, Xiaoguang Zhang, Hongchuan Wang, Chunchi Lei, Xiaobo Feng, Cao Yang, Yong Gao

**Affiliations:** 1 Department of Orthopaedics, Union Hospital, Tongji Medical College, Huazhong University of Science and Technology, Wuhan, 430022 China; 2 Department of Ophthalmology, Union Hospital, Tongji Medical College, Huazhong University of Science and Technology, Wuhan, 430022 China

**Correction to: Journal of Nanobiotechnology (2024) 22:31**

10.1186/s12951-023-02283-6

In this article **Yaqi He, Xin Liu, and Jie Lei** should have been denoted as equally contributing authors.

The original article [1] has been corrected.
