# Supplementary material for: Bioactive VS4-based sonosensitizer for robust chemodynamic, sonodynamic and osteogenic therapy of infected bone defects
Source: J Nanobiotechnology. 2024 Jan 16;22:31. doi: 10.1186/s12951-023-02283-6 (PMC10792985; doi:10.1186/s12951-023-02283-6)
Supplement: Supplementary file 1 — Additional file 1: Figure S1. Temperature changes during antibacterial treatment which was observed with a thermal imager. Figure S2. Antibacterial efficiency of materials after 6 months of placement. a Spread plate, b the number of MRSA colonies. Figure S3. Hydrogen Peroxide Assay Kit was used to test the H2O2 reduction rate, which was mainly benefit by the Pox-like activity of VS4. Figure S4. Materials morphology and EDS analysis.a TEM of VS4 nanorods; b TEM of MXene nanosheets. c EDS analysis of 3VSM. Figure S5. Characterization of nanoparticles. a Zeta potential of VS4, MXene and 3VSM. b The survey XPS spectra of VS4, MXene and 3VSM. c XPS spectrum in Ti 2p mode. d Raman spectra of VS4, MXene, and 3VSM. e Schottky junction mechanism. Figure S6. ARS staining of hBMSCs on day 14. [file 12951_2023_2283_MOESM1_ESM.docx]

Additional file 1

**Bioactive VS_4_-based** **sonosensitizer for robust chemodynamic, sonodynamic and osteogenic therapy of** **infected bone defects**

**Yaqi He^1^, Xin Liu^2^, Jie Lei^1^, Liang Ma^1^, Xiaoguang Zhang^1^, Hongchuan Wang^1^, Chunchi Lei^1^, Xiaobo Feng^1^, Cao Yang^1*^ and Yong Gao^1*^**

^1^ Department of Orthopaedics, Union Hospital, Tongji Medical College, Huazhong University of Science and Technology, 430022 Wuhan, China

^2^ Department of Ophthalmology, Union Hospital, Tongji Medical College, Huazhong University of Science and Technology, Wuhan 430022, China

^*^ Correspondence:

Cao Yang

caoyangunion@hust.edu.cn

Yong Gao

docgao@163.com

**
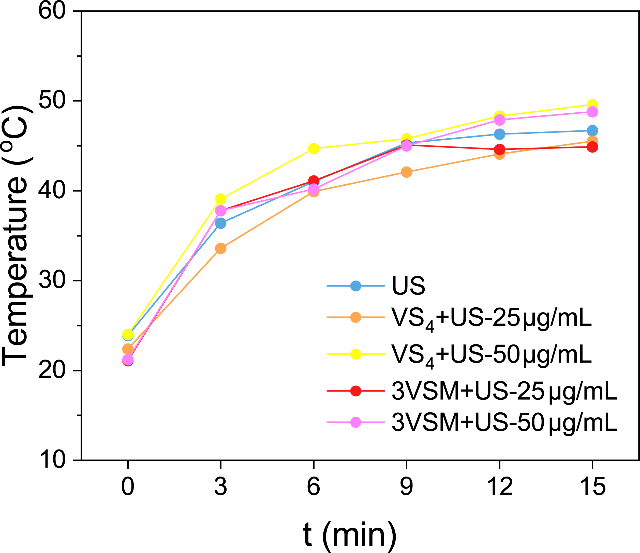
**

**Figure S1.** Temperature changes during antibacterial treatment which was observed with a thermal imager.


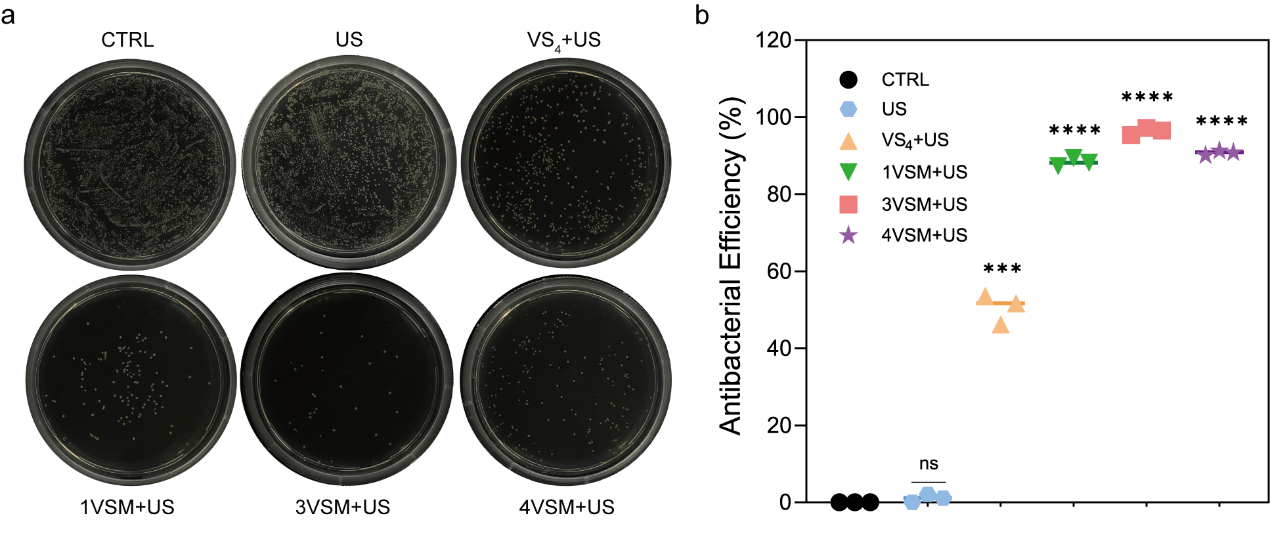


**Figure S2.** Antibacterial efficiency of materials after six months of placement. **a** Spread plate, **b** the number of MRSA colonies.

**
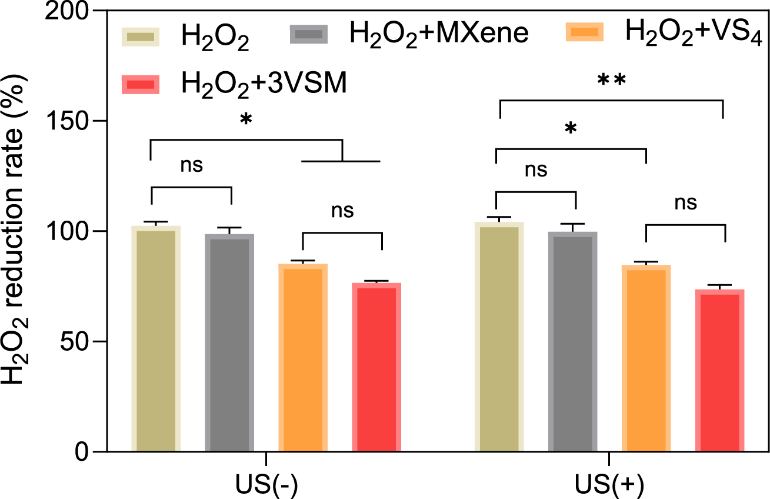
**

**Figure S3.** Hydrogen Peroxide Assay Kit was used to test the H_2_O_2_ reduction rate, which was mainly benefit by the Pox-like activity of VS_4_


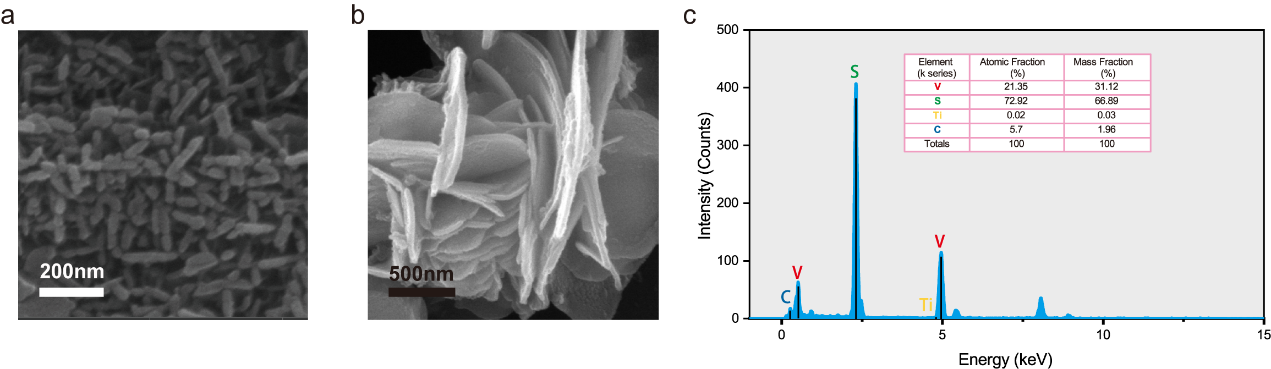


**Figure S4.** Materials morphology and EDS analysis. **a** TEM of VS_4_ nanorods; **b** TEM of MXene nanosheets. **c** EDS analysis of 3VSM


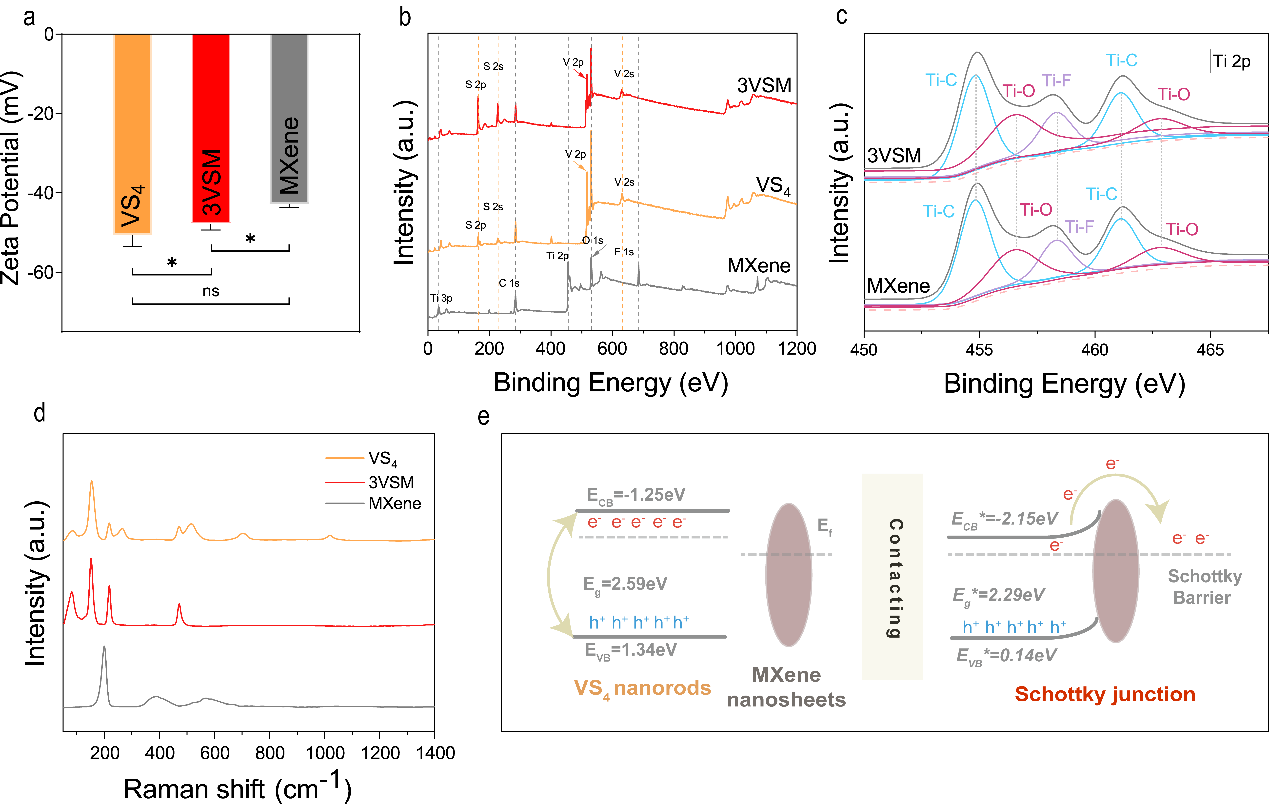


**Figure S5.** characterization of nanoparticles. **a** Zeta potential of VS_4_, MXene and 3VSM. **b** The survey XPS spectra of VS_4_, MXene and 3VSM. **c** XPS spectrum in Ti 2p mode. **d** Raman spectra of VS_4_, MXene, and 3VSM. **e** Schottky junction mechanism.


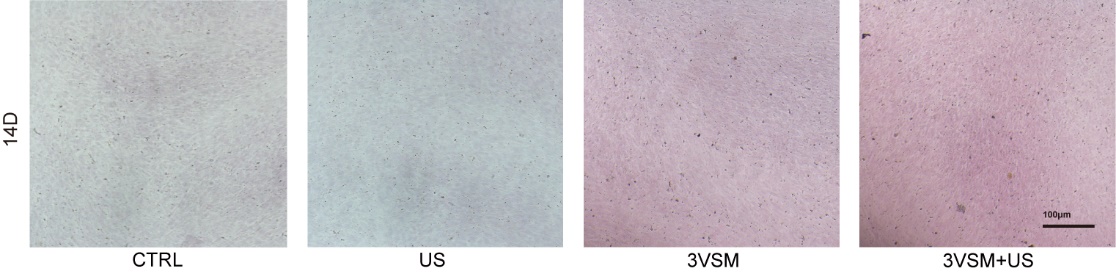


**Figure S6.** ARS staining of hBMSCs on day 14.
